# Comparison of Corneal Optical Quality After SMILE, Wavefront-Optimized LASIK and Topography-Guided LASIK for Myopia and Myopic Astigmatism

**DOI:** 10.3389/fmed.2022.870330

**Published:** 2022-04-05

**Authors:** Yu Zhang, Xiaoxiao Sun, Yueguo Chen

**Affiliations:** ^1^Department of Ophthalmology, Peking University Third Hospital, Beijing, China; ^2^Beijing Key Laboratory of Restoration of Damaged Ocular Nerve, Beijing, China

**Keywords:** SMILE, FS-LASIK, optical quality, topography-guided, wavefront-optimized, optical path difference, strehl ratio

## Abstract

**Purpose:**

To compare visual outcomes and corneal optical quality after small incision lenticule extraction (SMILE), wavefront-optimized (WFO) FS-LASIK, and topography-guided customized ablation treatment (TCAT) FS-LASIK for myopia.

**Methods:**

This prospective case-series study included 283 eyes of 283 myopic patients who underwent SMILE or FS-LASIK. There were 102, 100, and 81 eyes in the SMILE group, WFO group and TCAT group, respectively. The tomography system (Sirius) was used to measure corneal aberrations and optical quality.

**Results:**

At postoperative 1 and 6 months, there were no significant differences in uncorrected distance visual acuity and corrected distance visual acuity among the three groups (*P* > 0.05). Postoperative manifest refractive spherical equivalent was similar among the groups (*P* > 0.05). There was statistically significant difference in cylinder at 1 month among the three groups, with the highest mean value in TCAT group (*P* < 0.05). The corneal optical path difference, root mean square of corneal astigmatism and strehl ratio were the most superior in the TCAT group at postoperative 1 and 6 months (*P* < 0.05).

**Conclusion:**

SMILE, WFO FS-LASIK, and TCAT FS-LASIK provided similar visual results. The corneal visual quality after TCAT FS-LASIK was the best.

## Introduction

Small incision lenticule extraction (SMILE) and femtosecond laser-assisted LASIK (FS-LASIK) are two main stream laser surgical procedures of myopia and myopic astigmatism correction (1, 2). SMILE has been shown its potential advantages of reduced denervation, faster resolution of postoperative dry eye, and no flap-related risks (3–5). SMILE, however, relies on subjective fixation on a target light and has only one symmetric spherical ablation profile without eye tracking, iris registration and customized ablation profile. Several customized ablation algorithms of FS-LASIK have been developed. Wavefront-optimized (WFO) ablation attempts to reduce the induction of spherical aberration by adding peripheral pulses, blending it with the central ablation profile and maintaining the prolate shape of the cornea (6). Topography-guided customized ablation treatment (TCAT) attempts to maintain the aspheric shape of the cornea and neutralize corneal irregularities (7). There are some discussions on which of the two surgical methods is better for vision and visual quality. Some studies have found no significant difference between the two procedures (2, 8–12), whereas other studies have indicated that either SMILE or FS-LASIK should be preferred in terms of refractive results or higher-order aberrations (13–19).

Because the comparing results are still controversial, we conducted this prospective study comparing SMILE, WFO LASIK, and TCAT LASIK simultaneously regarding vision, refraction, corneal aberrations, and optical quality.

## Methods

### Patients

The present study was a prospective, non-randomized, comparative clinical study enrolling patients with myopia or myopic astigmatism. Two hundred and eighty-three eyes of 283 patients who underwent bilateral myopia and myopic astigmatism correction with FS-LASIK or SMILE from 2017 to 2019 were included in this prospective study. SMILE was performed in 102 patients (SMILE group). WFO FS-LASIK was performed in 100 patients (WFO group). TCAT FS-LASIK was performed in 81 patients (TCAT group). One eye of each patient was randomly chosen for analysis.

Inclusion criteria were age ≥18 years, myopic sphere up to 8.00 D, cylinder up to 1.50 D, with a documented refractive stability for a minimum period of 1 year and discontinuation of soft contact lenses for at least 2 weeks. Exclusion criteria included a residual stromal bed less than 280 μm, topographic evidence of corneal ectasia, previous ocular surgery, history of herpetic eye disease, collagen vascular disease, pregnancy, and lactation.

The study received approval from the Ethics Committee of Peking University Third Hospital and was conducted in accordance with the tenets of the Declaration of Helsinki. A written informed consent was obtained from each patient prior to the surgical procedure.

### Preoperative Examinations

Preoperative evaluation included uncorrected distance visual acuity (UDVA) and corrected distance visual acuity (CDVA), manifest refraction, slit lamp bio-microscopy, dilated fundus evaluation, corneal thickness (A scan, Tomey Japan), corneal tomography (Sirius, CSO, Italy) and corneal topography (Vario Topolyzer, WaveLight, Alcon Laboratories, Inc., Fort Worth, TX, United States). The total corneal aberrations and optical quality in a 6-mm zone were obtained from corneal tomography. Parameters of corneal aberrations and optical quality included optical path difference (OPD), root mean square of higher order aberration (RMSh), RMS of corneal astigmatism, RMS of spherical aberration (SA), RMS of coma, and strehl ratio (SR). Corneal topography data of kappa angle, static cyclotorsion compensation for excimer laser ablation and anterior corneal map for topography guided procedure were obtained from the Vario Topolyzer placido-based topography.

### Surgical Procedures

All surgeries were performed under topical anesthesia by an experienced refractive surgeon. For FS-LASIK, all flaps were created by the WaveLight FS200 femtosecond laser. The flap/canal/hinge parameters were as followed: flap thickness, 110 μm; flap diameter, 8.5–9.0 mm; side-cut angle, 90°; hinge angle, 50°; canal width, 1.5 mm. Following blunt dissection and flap lift, the stromal bed was ablated with excimer laser (EX500 WaveLight) using an optic zone of 6.5 mm with a 1.25 mm transition zone. The refraction data (sphere, cylinder, and axis) used for the eyes in the WFO group was the subjective manifest refraction. The refraction data used for the eyes in the TCAT group partially followed topography-modified refraction (TMR) scheme introduced by AJ Kanellopoulos (7).

SMILE was performed with the VisuMax femtosecond laser system (Zeiss, Germany, Nomogram version 3.0). The energy setting was 140 nJ, and the laser spot spacing was 4.5μm for the lenticule and cap interface, 2.0 μm for the lenticule side cut and cap side cut. The spherical refraction data was 10% more on the basis of manifest refraction. The lenticule base thickness was 10–15 μm. The cap thickness was 120μm, and the cap diameter was 7.6 mm with 2 mm small incision width. The lenticule optical zone (OZ) was 6.5 mm with 0.1 mm transition zone for astigmatism.

### Postoperative Care and Follow-Up

Postoperatively, all the eyes received treatment with 0.1% fluorometholone (FML, Allergan, Inc., Irvine, CA, United States) in tapering dose for 4 weeks, 0.5% levofloxacin (Cravit, Santen, Inc., Japan) four times a day for 2 weeks and lubricating drops four times a day for 4 weeks. Follow-up visits included postoperative day 1 and 7, month 1, 3, and 6. The follow-up examinations involved measurements of UDVA, slit-lamp examination, manifest refraction, CDVA and corneal tomography (Sirius, CSO, Italy). Corneal tomography was measured by the same technician who didn’t know the grouping of patients.

### Statistical Analysis

Data were analyzed using SPSS software (version 21.0; SPSS, Inc., Chicago, IL, United States). The Kolmogorov-Smirnov test was used for confirming normality of data. The normally distributed data were represented as mean ± standard deviation. Data not following normal distribution were presented as median (min, max). The normally distributed data were compared among the three groups using One-way ANOVA. *Post-hoc* multiple comparisons were performed between groups using Dunnett’s T3. Kruskal-Wallis H test was used to compare the non-normally distributed data among the three groups. If the Kruskal-Wallis test showed statistical significance, *post-hoc* pairwise comparisons were performed using Dunn-Bonferroni test. Comparisons of the distribution of visual acuity and refraction among the three groups were analyzed by Pearson chi-square test. A *P*-value of less than 0.05 was considered statistically significant.

## Results

This study included 283 eyes of 283 patients. There were 102 eyes, 100 eyes and 81 eyes in the SMILE group, WFO group and TCAT group, respectively. There was no statistically significant difference in the baseline data among the three groups regarding age, sex, preoperative refraction, corneal thickness, corneal aberrations, and Strehl ratio (*P* > 0.05) (Table 1). The mean maximal lenticule thickness or ablation depth were 109 ± 17 μm, 81 ± 19 μm and 90 ± 19 μm in the SMILE group, WFO group and TCAT group, respectively (*P* < 0.001). Thus, the mean thinnest corneal thickness were 442 ± 30 μm, 465 ± 33 μm, and 458 ± 29 μm at 6 months, respectively (*P* < 0.001). All patients completed 6-month follow-ups.

**TABLE 1:** Baseline clinical data and demographics in the three groups.

	SMILE (*n* = 102)	WFO (*n* = 100)	TCAT (*n* = 81)	F/χ^2^	*P*
Sex (male/female)	22/80	24/76	15/66	0.795	0.672
Age (year)	28.2 ± 6.1	29.9 ± 7.2	29.5 ± 6.7	1.929	0.147
Sphere (D)	−5.09 ± 1.26	−4.95 ± 1.57	−5.22 ± 1.49	0.754	0.471
Cylinder (D)	−0.63 ± 0.31	−0.63 ± 0.38	−0.65 ± 0.37	0.110	0.896
ThkMin (μm)	548 ± 24	544 ± 31	545 ± 26	0.623	0.538
OPD (μm)	0.88 ± 0.35	0.93 ± 0.41	0.98 ± 0.39	1.550	0.214
RMSh (μm)	0.41 ± 0.13	0.42 ± 0.13	0.44 ± 0.15	0.763	0.467
RMS-AST (μm)	0.75 ± 0.40	0.80 ± 0.45	0.86 ± 0.42	1.527	0.219
RMS-coma (μm)	0.22 ± 0.13	0.22 ± 0.13	0.25 ± 0.15	1.417	0.244
RMS-SA (μm)	0.22 ± 0.07	0.22 ± 0.07	0.23 ± 0.08	0.683	0.506
Strehl ratio	0.14 (0.06, 0.72)	0.15 (0.05, 0.32)	0.15 (0.06, 0.27)	0.844	0.656

*SMILE, small incision lenticule extraction; WFO, Wavefront-optimized laser in situ keratomileusis; TCAT, Topography-guided custom laser in situ keratomileusis; ThkMin, thinnest corneal thickness; OPD, optical path difference; RMSh, root mean square of higher order aberrations; AST, corneal astigmatism; SA, spherical aberration.*

### Uncorrected Distance Visual Acuity Outcomes

At postoperative 1 month, UDVA of 20/20 or better was measured in 95.1% of eyes in the SMILE group, 97.0% of eyes in the WFO group and 93.8% of eyes in the TCAT group (*P* = 0.393).

**FIGURE 1:**
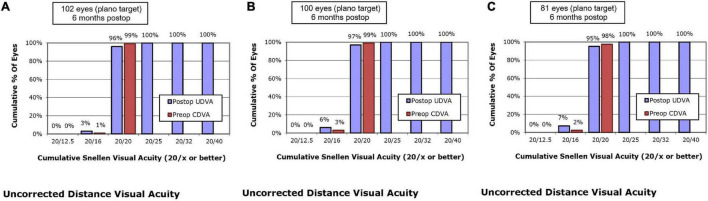
Postoperative 6-month uncorrected distance visual acuity vs. preoperative corrected distance visual acuity. **(A)** SMILE group, **(B)** wavefront optimized group, **(C)** topography-guided custom ablation treatment group.

At postoperative 6 months, UDVA of 20/20 or better was measured in 96.1% of eyes in the SMILE group, 97.0% of eyes in the WFO group and 95.1% of eyes in the TCAT group (*P* = 0.658) (Figure 1).

### Corrected Distance Visual Acuity Outcomes

At postoperative 1 month, 10.8% of the eyes in the SMILE group, 17.0% in the WFO group and 9.9% in the TCAT group gained one line of CDVA. A loss of one line was noted in 5.9, 7.0, and 9.9% of the eyes in the SMILE, WFO and TCAT groups, respectively. No change in line was noted in 78.4, 61.0, and 71.6% of the eyes in the SMILE, WFO, and TCAT groups, respectively (*P* = 0.083).

**FIGURE 2:**
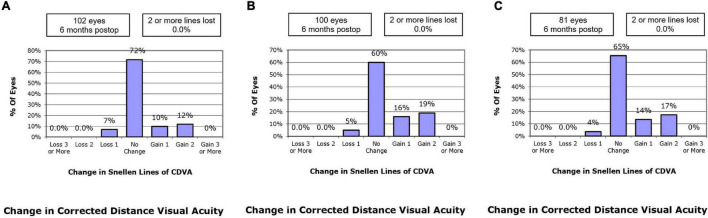
Change in Snellen lines of corrected distance visual acuity at postoperative 6-month. **(A)** SMILE group, **(B)** wavefront optimized group, **(C)** topography-guided custom ablation treatment group.

At postoperative 6 months, 9.8% of the eyes in the SMILE group, 16.0% in the WFO group and 13.6% in the TCAT group gained one line of CDVA. A loss of one line was noted in 6.9, 5.0, and 3.7% of the eyes in the SMILE, WFO, and TCAT groups, respectively. No change in line was noted in 71.6, 60.0, and 65.4% of the eyes in the SMILE, WFO, and TCAT groups, respectively (*P* = 0.501) (Figure 2).

### Refractive Outcomes

Postoperative MRSE and cylinder at 1 and 6 months are shown in Table 2. There was statistically significant difference in cylinder at 1 month among the three groups (*P* < 0.05). Meanwhile, the cylinder at 1 month in the WFO group was significant less than that in the TCAT group (*P* < 0.05).

**TABLE 2:** Comparison of postoperative manifest refraction among the three groups.

	SMILE (*n* = 102)	WFO (*n* = 100)	TCAT (*n* = 81)	χ ^2^	*P*
MRSE 1M (D)	0 (−0.75, 0.75)	0 (−1.00, 1.00)	0 (−1.25, 1.13)	5.293	0.071
MRSE 6M (D)	0 (−0.75, 0.63)	0 (−0.63, 0.88)	0 (−0.63, 1.00)	3.445	0.179
Cylinder 1M (D)	−0.25 (−1.25, 0)	0 (−1.00, 0)*	−0.25 (−1.00, 0)*	6.655	0.036
Cylinder 6M (D)	0 (−1.00, 0)	0 (−1.00, 0)	−0.25 (−1.00, 0)	1.959	0.375

*The value was significantly different between the two groups.

*SMILE, small incision lenticule extraction; WFO, Wavefront-optimized laser in situ keratomileusis; TCAT, Topography-guided custom laser in situ keratomileusis; MRSE, manifest refractive spherical equivalent.*

**FIGURE 3:**
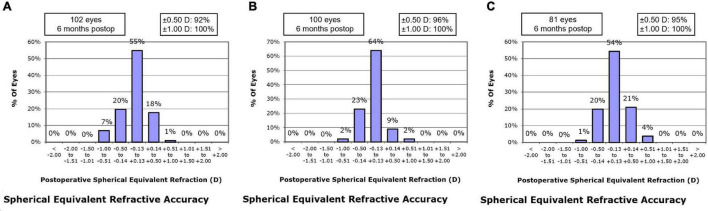
Spherical equivalent refractive accuracy at postoperative 6-month. **(A)** SMILE group, **(B)** wavefront optimized group, **(C)** topography-guided custom ablation treatment group.

At postoperative 1 month, the residual MRSE within ± 0.13 D was achieved by 55.9% of eyes in the SMILE group, 55.0% of eyes in the WFO group and 48.1% of eyes in the TCAT group (*P* = 0.543). At postoperative 6 months, the residual MRSE within ± 0.13 D was achieved by 54.9% of eyes in the SMILE group, 64.0% of eyes in the WFO group and 54.3% of eyes in the TCAT group (*P* = 0.127) (Figure 3).

**FIGURE 4:**
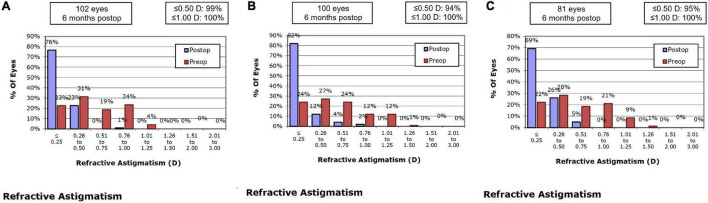
Refractive astigmatism accuracy at postoperative 6-month. **(A)** SMILE group, **(B)** wavefront optimized group, **(C)** topography-guided custom ablation treatment group.

At 1 month, 76.5% of eyes in the SMILE group, 76.0% of eyes in the WFO group and 65.4% of eyes in the TCAT group showed refractive astigmatism of 0.25 D or less. There was no statistically significant difference in refractive astigmatism among the three groups (*P* = 0.095). At 6 months, 76.5% of eyes in the SMILE group, 82.0% of eyes in the WFO group and 69.2% of eyes in the TCAT group showed refractive astigmatism of 0.25 D or less (*P* = 0.055) (Figure 4).

### Corneal Aberrations and Optical Quality

At both 1 and 6 months, OPD and RMS of corneal astigmatism in the TCAT group were significantly lower than the other two groups (*P* < 0.05). However, there were no statistically significant differences in RMSh, RMS of coma or SA among the three groups (*P* > 0.05). At postoperative 1 month, SR in the TCAT group was significantly higher than that in the other two groups (*P* < 0.05). At 6 months, SR was significantly different among the three groups (*P* < 0.05), and SR in the SMILE group was significantly less than the other two groups (*P* < 0.05) (Tables 3, 4).

**TABLE 3:** Comparison of corneal aberrations and optical quality among the three groups at 1 month postoperatively.

	SMILE (*n* = 102)	WFO (*n* = 100)	TCAT (*n* = 81)	F	*P*
OPD (μm)	0.92 ± 0.40	0.90 ± 0.31	0.77 ± 0.28*	5.028	0.007
RMSh (μm)	0.67 ± 0.26	0.69 ± 0.25	0.67 ± 0.27	0.305	0.738
RMS-AST (μm)	0.57 ± 0.41	0.52 ± 0.30	0.33 ± 0.19*	13.450	0.000
RMS-coma (μm)	0.38 ± 0.22	0.38 ± 0.21	0.34 ± 0.21	1.178	0.309
RMS-SA (μm)	0.42 ± 0.16	0.45 ± 0.21	0.44 ± 0.20	0.681	0.507
Strehl ratio	0.16 ± 0.04	0.16 ± 0.04	0.19 ± 0.05*	12.938	0.000

*The value was significantly different from that in the other two groups.

*SMILE, small incision lenticule extraction; WFO, Wavefront-optimized laser in situ keratomileusis; TCAT, Topography-guided custom laser in situ keratomileusis; OPD, optical path difference; RMSh, root mean square of higher order aberrations; AST, corneal astigmatism; SA, spherical aberration.*

**TABLE 4:** Comparison of corneal aberrations and optical quality among the three groups at 6 months postoperatively.

	SMILE (*n* = 102)	WFO (*n* = 100)	TCAT (*n* = 81)	F	P
OPD (μm)	0.91 ± 0.26	0.93 ± 0.29	0.81 ± 0.27*	5.078	0.007
RMSh (μm)	0.70 ± 0.20	0.73 ± 0.23	0.71 ± 0.27	0.462	0.631
RMS-AST (μm)	0.53 ± 0.30	0.52 ± 0.30	0.34 ± 0.19*	13.547	0.000
RMS-coma (μm)	0.40 ± 0.20	0.40 ± 0.22	0.38 ± 0.22	0.330	0.719
RMS-SA (μm)	0.45 ± 0.16	0.47 ± 0.19	0.47 ± 0.20	0.414	0.662
Strehl ratio	0.16 ± 0.04*	0.17 ± 0.06	0.19 ± 0.05	9.450	0.000

*The value was significantly different from that in the other two groups.

*SMILE, small incision lenticule extraction; WFO, Wavefront-optimized laser in situ keratomileusis; TCAT, Topography-guided custom laser in situ keratomileusis; OPD, optical path difference; RMSh, root mean square of higher order aberrations; AST, corneal astigmatism; SA, spherical aberration.*

### Complications

All surgeries were successfully completed and no serious intraoperative and postoperative complications occurred. Two eyes in the WFO group and one eye in the TCAT group had mild DLK in the first week after operation. Slight corneal interface edema occurred in two eyes in the SMILE group because of difficult separation of the lenticule. No patients needed re-treatment because of unsatisfactory vision or complications.

## Discussion

The current study found no significant differences in postoperative UDVA or CDVA among SMILE, WFO, and TCAT groups, indicating that the three procedures were comparably effective and safe. The majority of previous studies demonstrated the similar results (2, 8–15, 17–19). A randomized, paired-eye study found that SMILE achieved similar results to WFO LASIK in terms of efficacy index (0.97 ± 0.20 vs. 0.99 ± 0.20; *P* = 0.56), UDVA of 20/40 or better (100 vs. 100%; *P* = 1.0), and UDVA of 20/20 or better (84 vs. 87%; *P* = 0.63) (9). However, a prospective, randomized contralateral eye study found that 86.4% of the topography-guided LASIK group and 68.2% of the SMILE group had UDVA of 20/20 (*P* < 0.002) and 59.1 and 31.8%, respectively, had UDVA of 20/16 (*P* < 0.002) at 3 months (16). The inferior results of SMILE may be due to a steeper surgeon learning curve of SMILE, which may cause inferior visual outcomes during the early stage of operations (20). The surgeon of the present study had more than 1 year experience of performing SMILE, so SMILE group showed good visual results similar to WFO group and TCAT group.

The present study found that postoperative refractive results at 1 and 6 months were similar among the three groups, except that the 1-month postoperative cylinder was significantly different among the three groups, and more residual cylinder was noted in the TCAT group than WFO group (*P* < 0.05). Most previous studies showed comparable refractive results between SMILE and FS-LASIK (2, 8–15, 18, 19, 21). The high residual cylinder in the TCAT group of the present study was similar to our previous study (22), which might be caused by the compensation method for TCAT design. In the present study, partial TMR was applied to the TCAT design of cylinder and axis. However, Kanellopoulos AJ (16) compared SMILE and Topo-LASIK with TMR method and found that Topo LASIK was superior in postoperative MRSE and cylinder to SMILE. The higher residual refraction in SMILE might be caused by inappropriate nomogram of parameter design, high preoperative cylinder, lack of eye tracking and cyclotorsion compensation. Better design methods for TCAT are needed to obtain more accurate refractive results. The Phorcides Analytic Engine for topography-guided surgery planning was preliminarily proved to have good refractive results, with 83% of eyes showing a refractive cylinder of less than 0.25 D postoperatively (23). Interestingly, 6-month postoperative cylinder in TCAT group decreased and was similar to the other groups. We speculate that the astigmatism change was due to the compensative morphological change of the lens, which need further verification in the future.

Most aberrations come from the cornea. Good corneal optical quality is the premise of the visual quality. The present study also found that corneal OPD, RMS of corneal astigmatism and SR in the TCAT group were the most superior, showing that corneal optical quality after TCAT LASIK was the best. To the best of our knowledge, no previous study compared the corneal aberrations and optical qualities among the three procedures. The lowest OPD in the TCAT group mainly derived from the lowest corneal astigmatism, but not from the higher-order aberrations. Several studies showed lower corneal coma after wavefront-guided LASIK compared to SMILE, which might be due to automatic eye tracking with active centration control and cyclotorsion compensation in LASIK (14, 19). In the present study, however, RMS of coma was comparably low in the TCAT group, but the difference wasn’t statistically significant. On the contrary, Yin Y et al. found lower coma aberration at 1 month after SMILE than FS-LASIK, which may be due to high myopic correction and iTrace analyzer measurement (24). Some studies also found lower corneal SA after SMILE compared to FS-LASIK (13–15). As we known, SMILE procedure could achieve a larger functional optical zone than do FS-LASIK procedures because of less biomechanical alterations in the peripheral area of the cornea after SMILE (25, 26). Postoperative spherical aberration was associated with the area of functional optical zone (27, 28). Yet, in the present study, postoperative SA in the SMILE group was comparably low, but compared with the other two groups, there was no significant difference. Similar to the present study, El-Mayah et al. also found a similar comparative trend of postoperative coma and SA between SMILE and FS-LASIK (*P* > 0.05) (12). In the future, larger sample, randomized controlled studies are needed to further compare corneal aberrations and optical quality among the three surgical procedures.

In our previous study, we found similar visual and refractive results between WFO and TCAT groups, and higher corneal optical quality in the TCAT group (22). The purpose of the present study is to compare SMILE and FS-LASIK. WFO and TCAT are two common customized ablation profiles of FS-LASIK, so SMILE was compared with WFO and TCAT at the same time in the present study. We found SMILE was almost similar to WFO LASIK in terms of visual result, refractive outcomes and corneal optical quality. However, SMILE provided inferior outcomes in corneal optical quality to TCAT LASIK. Thus, the comparison of corneal optical quality between SMILE and FS-LASIK depends on the different ablation profiles, which can’t be generalized.

The limitations of the present study are as follows. First, we only compared visual acuity, refraction, corneal aberrations and optical qualities among the three groups, and other examinations related with visual quality, such as contrast-sensitivity function, objective scatter index, and questionnaires regarding glare and halo weren’t studied. Second, the study had not a randomized sample and was subject to selection bias that might result in an unbalanced selection of patients. Third, we only included myopic subjects with low astigmatism (<1.50 D). Future randomized studies with more parameters of optical quality and greater astigmatism range are needed.

In conclusion, SMILE, WFO FS-LASIK and TCAT FS-LASIK provided similar visual and refractive results. TCAT FS-LASIK could induce fewer corneal optical path difference and astigmatism, and higher strehl ratio than the others. However, TCAT FS-LASIK could induce more manifest residual cylinder and a more accurate algorithm for compensating the irregular ablation, corneal posterior surface and internal eye astigmatism is needed to further improve postoperative visual acuity and refractive outcomes.

## Data Availability Statement

The raw data supporting the conclusions of this article will be made available by the authors, without undue reservation.

## Ethics Statement

The studies involving human participants were reviewed and approved by the Ethics Committee of the Peking University Third Hospital. The patients/participants provided their written informed consent to participate in this study.

## Author Contributions

YZ conducted the statistical analysis and wrote the manuscript. XS collected the data. YC designed the study and supervised the project. All authors contributed to the article and approved the submitted version.

## Conflict of Interest

The authors declare that the research was conducted in the absence of any commercial or financial relationships that could be construed as a potential conflict of interest.

## Publisher’s Note

All claims expressed in this article are solely those of the authors and do not necessarily represent those of their affiliated organizations, or those of the publisher, the editors and the reviewers. Any product that may be evaluated in this article, or claim that may be made by its manufacturer, is not guaranteed or endorsed by the publisher.
